# Production Efficiency and Market Orientation in Food Crops in North West Ethiopia: Application of Matching Technique for Impact Assessment

**DOI:** 10.1371/journal.pone.0158454

**Published:** 2016-07-08

**Authors:** Habtamu Yesigat Ayenew

**Affiliations:** Production and Resource Economics, Technical University Munich, Freising, Germany; University of the Basque Country, SPAIN

## Abstract

**Introduction:**

Agricultural technologies developed by national and international research institutions were not benefiting the rural population of Ethiopia to the extent desired. As a response, integrated agricultural extension approaches are proposed as a key strategy to transform the smallholder farming sector. Improving Productivity and Market Success (IPMS) of Ethiopian Farmers project is one of the development projects initiated by integrating productivity enhancement technological schemes with market development model. This paper explores the impact of the project intervention in the smallholder farmers’ wellbeing.

**Methods:**

To test the research hypothesis of whether the project brought a significant change in the input use, marketed surplus, efficiency and income of farm households, we use a cross-section data from 200 smallholder farmers in Northwest Ethiopia, collected through multi-stage sampling procedure. To control for self-selection from observable characteristics of the farm households, we employ Propensity Score Matching (PSM). We finally use Data Envelopment Analysis (DEA) techniques to estimate technical efficiency of farm households.

**Results:**

The outcome of the research is in line with the premises that the participation of the household in the IPMS project improves purchased input use, marketed surplus, efficiency of farms and the overall gain from farming. The participant households on average employ more purchased agricultural inputs and gain higher gross margin from the production activities as compared to the non-participant households. The non-participant households on average supply less output (measured both in monetary terms and proportion of total produce) to the market as compared to their participant counterparts. Except for the technical efficiency of production in potato, project participant households are better-off in production efficiency compared with the non-participant counterparts.

**Conclusion:**

We verified the idea that Improving Productivity and Market Success (IPMS) of Ethiopian farmers’ project has contributed for the input and out market integration and/or market oriented agricultural production. Overall, we argue that these can be seen as an experimental model with a promising potential to improve the livelihood of the poor. Furthermore, we suggest that it is worthwhile to employ integrated agricultural extension programs with further targeting in the developing world.

## Introduction

The concept of market orientation in agriculture is rather a common phrase in the developing and transition economies policy discussions and literature [1, 2]. Oftentimes, the transformation of the subsistence sector to market oriented agribusiness model is seen as the key for development in sub-Saharan Africa [2]. In sub-Saharan Africa, the weakly integrated agrarian setup with the market economy in smallholder context has become the essential element of the market oriented transformation process [3]. Nonetheless, finding the right approach of transformation that can guarantee improvement in smallholders’ wellbeing, and having the necessary infrastructure and institutions to support the process remain crucial questions to address.

The economy of Ethiopia is predominantly agrarian, where smallholders contribute about 90% of agricultural production [4]. Improving the contribution of the sector in the whole economy can’t be realized without improved adoption of production technologies and the actual participation of smallholders. By realizing this fact, successive strategy papers have been developed as part of the Agriculture Development Led Industrialization (ADLI) policy in Ethiopia emphasizing the importance of market orientation in smallholder agriculture. the public extension system is given the crucial role in technology dissemination and knowledge transfer to smallholder farmers [4, 5]. Despite a general consensus that the sector’s transformation to market oriented production model is the way out of poverty [2], little progress has been achieved so far. For instance, Mengistu [6] highlighted that the sector in the country is less efficient both in terms of land and labor. Resource poor rural households with lower productivity migrate to urban areas in search of livelihood options [7].

The public agricultural extension system is organized with units extending to the smallest administrative unit to facilitate technology transfer process to improve production and productivity of the sector [8]. Nonetheless, the adoption of improved technologies remained apparently low [9–12]. The global experience also revealed that even for beneficial technologies, many years might have passed without being used or might not also be used at all by farmers [13]. The little fit of features of technologies themselves to farmers and their social and cultural environment, human capital and other resource constraints, input and output market organization and institutional and political factors might contribute to the low rate of adoption of technologies [6, 9, 10]. The overall implication of the transformation strategy can also be influenced through the organizational aspects and approaches of the system itself [2]. This makes more sense in countries like Ethiopia, where smallholder farmers are sparsely located in areas with poor infrastructure, with little linkage to the market, and with long history of traditional and subsistence farming system. Owing to both the micro-economic elements and poor organizational support, the available technologies were not benefiting the rural population of Ethiopia to the extent desired [6, 9, 10, 12].

Different governmental and non-governmental projects have been working in the sector to support the extension system. Improving Productivity and Market Success (IPMS) of Ethiopian Farmers project is one of these projects initiated by integrating productivity enhancement technological schemes with market development model. Since 2007, IPMS project has been working in some selected districts of Ethiopia, and Bure district is one of the model sites to implement the integrated extension approach. The project aims to improve the livelihood of farmers though improved access to agricultural technologies, trainings on value addition techniques and improved product quality, input and output market linkage and improved access to credit [14]. To the broader sense, it is vital to answer if the used approach brings about a significant improvement on market oriented production, efficiency of farms and farmers livelihood.

## Theoretical Model

The research hypothesis in this paper is to evaluate if project participation brought a change in the economic participation and livelihood of farm households. The integrated agricultural extension approach based intervention by the project is expected to improve social capital [15], efficiency and productivity [16], market orientation and market supply of households [14]. The impact of the project can be evaluated by enumerating the change in the participant households’ outcomes after the participation in the project. The basic idea of impact assessment tools is trying to figure out the difference in the outcomes of the household after the participation in the project.

Let’s denote the vector of outcome variables with Y, where Y_i(1)_ and Y_i(0)_ represent the potential outcomes of the households in the treatment and control groups respectively. The treatment effect can be calculated as the difference of the two outcomes.

$$TE_{i}=\;Y_{i\left(1\right)}\;-Y_{i\left(0\right)}$$ (1)

The major challenge in this case is a farm household can either be a participant or non-participant of the project activities. Since the household can’t be observed in both states, we encounter a missing data problem [16]. A couple of statistical solutions are proposed to solve such a fundamental problem in non-experimental data. Propensity Score Matching (we call PSM hereafter) technique is one of them introduced in literature to match households from the treatment and control groups using propensity scores [16, 17]. A household could participate in the project activities or decide otherwise by comparing the expected utility gain from the participation decision. From the utility maximization framework, we know that a household will participate in the project if the expected return from participation is higher than the existing level. This can be expressed as:

$$P_{i}=\{\begin{array}{ll} 1 & if\;\left(U_{i}^{p}-U_{i}^{np}\right)>0\\ 0 & if\;\left(U_{i}^{p}-U_{i}^{np}\right)\leq 0 \end{array}$$ (2)

where U_i_^p^ is the expected utility gain from participation in the project and U_i_^np^ is the expected utility from non-participation in the project and is (P_i_) the probability of participation of the household i. However, it is difficult to estimate this equation in the existing form since utility of the household is unknown. In addition, the farm household is unable to a priori observe the outcome before the participation decision. The PSM technique enables the researcher to calculate this probability as a function of observed covariates that can determine the participation decision.

In PSM statistical tool, given X is a vector of the exogenous variables that influence the decision to participate, matching can be performed conditioning on p(X) rather than on X alone. p(X) = probability (D = 1/X) is the probability of participating conditional on X, which finally is the propensity score of X [17]. In other words, PSM matches each participant household with a non-participant household that has (almost) the same likelihood of participating into the program to calculate the treatment effect [16]. With this simplifying assumption, the probability of participation in the project can be estimated with a function:

$$P_{i}^{*}=\beta X_{i}+\varepsilon_{i}$$ (3)

Where Xi are explanatory variables which influence the participation decision of the household in the project, *ε*_*i*_ is the random noise in the estimation.

## Description of the IPMS Project and the Dataset

Bure is one of the 15 districts of West Gojam Administrative. The district has moist and wet lowland (10%), wet mid altitude (82%) and wet high altitude (8%), and is one of the productive areas in the region. The altitude of the district ranges from 713 to 2604 meters above sea level (masl). Temperature of the study area ranges from 14 to 24 with the mean annual temperature of about 19°c [18]. The total area of the district is 72,739 ha, of which 46.6% is cultivated. The proportion of the landmass under forestland is 8.4% and the area under natural pasture is 6.0%. Since common grazing area and crop residue are the main sources of livestock feed in the district, feed shortage is among critical challenges influencing the productivity of the system. The average cultivated land holding of in the area is about 1.6 ha. [18].

### Description of the IPMS project

IPMS adopt pilot learning approaches to develop the capacity of smallholders through trainings, experience sharing visits, and institutional arrangements in rural areas [14]. In the commodity development work of the project, wheat, pepper, bean, potato, sugarcane, apple, mango, papaya, avocado, banana, honey production, sheep and cattle fattening enterprises are selected as the intervention commodities. With these commodities, the project has been trying to enhance the knowledge, uptake and use of technological and institutional innovation, information and knowledge management for market development. IPMS project provides assistance to the farmers to integrate themselves to the market and hence adjust the production and marketing decisions with market signals [14]. There was no criteria by the project to select participants and farmers join the project with their self-interest.

### Data and survey design

Both primary and secondary data sources were consulted for the study. We use secondary data about the district that was collected for and summarized in the diagnostic survey report of the district in 2007 [18]. Eight Focus Group Discussions (FGD) were organized at the start and end of the collection of data through formal questionnaires. Each FGD include 8–10 members of the society (elders, society and religious leaders, housewives etc.), who are participants and non-participants of the project. These FGDs were especially relevant to fully understand the intervention, the society and the area as well. Cross-section data were collected in 2010 through semi-structured questionnaires, using multi-stage sampling procedure and probability proportionate to size technique for the purpose. The project has intervened in some selected peasant associations (locally known as *Kebeles*), and four of them were randomly included in the sample for the collection of data. 100 project participant and 100 non participant farmers were interviewed using trained enumerators. In the questionnaire, recalling method were employed to get some socio-economic, demographic, institutional and organizational aspects before the intervention of the project, and hence used for the estimation of propensity scores. Our dataset comprises of both male headed (80%) and female headed (20%) farm households: The mean landholding of the farm households is 1.33 hectares. A significant proportion of crop harvest comes from rain fed agriculture, and only a small proportion of the land has access to irrigation water. We report the overall descriptive statistics in Table 1.

**Table 1:** Summary statistics of the dataset.

Variables	Total Sample	Participant	Control	Diff.
	Mean (Std. dev)	Mean (Std. dev)	Mean (Std. dev)	(t-)
Age of the household head(years completed)	44.03 (11.97)	43.23 (13.32)	44.82 (10.46)	-0.94
Sex of the head (dummy = 1 if women and, 0 otherwise)	0.20 (0.40)	0.24 (0.43)	0.16 (0.37)	1.41
Family size	6.10 (1.99)	6.37 (1.94)	5.84 (2.02)	1.89*
Community role (dummy = 1 if has role and, 0 otherwise)	0.59 (0.49)	0.70 (0.50)	0.47 (0.46)	3.38***
Literacy (dummy1 if literate and, 0 otherwise)	0.41 (0.49)	0.43 (0.49)	0.39 (0.49)	0.57
Livestock holding (in TLU)	5.85 (4.16)	6.78 (4.02)	4.93 (4.12)	3.21***
Landholding (in hectare)	1.33 (0.94)	1.52 (1.08)	1.17 (0.72)	2.63***
Irrigated landholding (in hectare)	0.08 (0.15)	0.08 (0.01)	0.08 (0.02)	0.10
Time to extension office (in hours)	0.44 (0.35)	0.46 (0.35)	0.42 (0.35)	-0.68
Time to market (in hours)	1.28 (0.60)	1.23 (0.68)	1.34 (0.51)	-0.81
Time to town (in hours)	1.70 (0.90)	1.65 (0.91)	1.75 (0.89)	-0.83

Source: Author’s Survey, 2010

*** and *means significant at the 1 and 10% probability levels, respectively.

The results of descriptive statistics of the explanatory variables show that there are statistically significant differences between the two groups with respect to some of the exogenous variables. Participants and non-participants of the project have significant differences on family size, landholding, livestock ownership and in the engagement community leadership. This might indicate the possible non-random selection of farm households towards participation in the project.

### Ethical statements

The study used survey data (anonymous) collected using questionnaires and ethical statements are not required for such kinds of studies. After explaining how the researcher will use the information, participants were asked if they are willing to participate they provide their verbal informed consent. Most of the participants were illiterate; it was not possible to take a written consent. In addition, in the study area, it is common to take a verbal consent. Furthermore, the ethics committee of the Department of Agricultural Economics of Haramaya University provided a statement of exemption for the present study. Confidentiality of information was ensured through avoiding any personal identifiers from data collection tools, proper handling of data and all other methods.

## Estimation Strategy

The first step in PSM is the estimation of the propensity scores. We employ the Logit model to estimate propensity scores for each observation in the sample. The dependent variable in the logit model was participation in the project, which took the value of 1 if a household is a participant and 0 otherwise. The explanatory variables were of different factors that determine the participation decision of the household in the project.

Following eq (3), the mathematical formulation of logit model is as follows:

$$P_{i}=\frac{e^{Z_{i}}}{\left(1+Z_{i}\right)}$$ (4)

Where P_i_ = 1 if a household is a participant and 0 otherwise

$$Z_{i}=\alpha_{0}+\sum \alpha_{i}X_{i}+U_{i}\;i=\;1,\;2,\;3,\;-\;--,\;n,$$ (5)

*α*_0_ is the intercept, α_i *=*_ regression coefficients to be estimated, *X*_*i*_ are pre-intervention explanatory variables like age, family size, level of education, land size, livestock etc., *U*_*i*_ is the disturbance term. The probability that a household belongs to the non-participant group is:

$$1-P_{i}=\frac{1}{\left(1+e^{Z_{i}}\right)}$$ (6)

There are some empirical challenges that one has to deal with in PSM. The use of the Logit model to estimate the propensity scores is based on the assumption that the researcher can observe these covariates (unconfoundedness assumption) [17, 19]. First, covariates used in the model have critical importance and should be selected properly. In this research, explanatory variables were selected based on findings of prior works on the issue and the informal survey done prior to the actual survey work. Second, the estimation result might be inconsistent if unobservable characteristics determine the participation decision of the household [17, 19, 20].

The main interest of the research is to calculate the average treatment effect on the treated and it is important to note that they are only defined in the region of common support. Hence, an important step is to check the overlap and the region of common support between treatment and comparison group. Implementing the common support condition ensures that any combination of characteristics observed in the treatment group can also be observed among the control group [21]. Treatment effect cannot be estimated for individuals that fall outside this region (those below the minima and above the maxima) [21, 22]. The next step in PSM is to select a matching algorithm that best fits with the nature of data. The matching algorithm matches observations from the participant and the control group based on the propensity scores. Nearest neighbor matching, caliper matching and kernel matching are most commonly used in empirics in PSM [20, 22–24]. It is quite important to keep in mind that there is no “theoretically best” matching algorithm superior than others and all of them have strengths and weaknesses.

The final procedure in PSM is estimation of the treatment effect. It is important to note that the variances and standard errors should be corrected. This is particularly relevant because the estimation of propensity scores, inclusion of only the common support region and the order the peers are matched could affect the variance. The widely applied procedure to correct the variance in PSM is bootstrapping technique, which brings the distribution of the matched sample standard error closer (approximately the same) to the population standard error [22, 24]. The treatment effect is evaluated for the use of purchased inputs, value of market supply, the share of product sold, gross margin and the technical efficiency difference among the participant and non-participant farmers.

Given the advantage that no prior functional form assumption is required by the approach [25, 26], Data Envelopment Analysis was employed to analyze technical efficiency. The DEA model with agricultural inputs (land, labor and cost for inputs used for production of the major food crops aggregated in monetary terms) and 4 outputs majorly produced in the area (wheat, potato, beans and pepper) is employed. Using the inputs and outputs under consideration, it develops a piece-wise production frontier and then estimates efficiency of farms. The main challenge here is since not all the households produce all the commodities mentioned, the analysis is done for one commodity at a time. Mathematically, the input oriented DEA model is defined as [26]:

$$\begin{array}{l} TE\left(X_{ki},Y_{mi}\right)=\underset{\theta ,\lambda }{\text{min}}\theta ,\left(\theta ,X_{ki},Y_{mi}\right)\\ subject\;to\;\left\{ \begin{array}{l} y_{m}{}_{i}\leq \lambda_{i}y_{m},\\ \sum_{i=1}^{I}\lambda_{i}x_{k}\leq \theta x_{ki},k=1,2\ldots k\\ \lambda_{i}\geq 0,i=1,2,\ldots I \end{array}\right. \end{array}$$ (7)

Where θ_i_—technical efficiency estimate to be calculated for each farm household i,

*Y*_mi_—quantity of output m produced by farm household i

*x*_ki_—quantity of input k used by household i

*λ*_i_—weight for household i

With the general premise that participation in the project (technology, knowledge, training, input and output market linkage) will improve the efficiency of the household and farm operation, we have conducted this study. The outcome of the efficiency analysis is then used to compare if there exists efficiency difference among the participant and non-participant households in the project. The technical efficiency, based on the aforementioned approach was analyzed using DEAP version 2.1 [26, 27].

Sensitivity analysis finally employed since the overall estimation in PSM is inherently based on unconfoundedness assumption. This assumption is often cited as a strong assumption in behavioral economics context [19, 28]. To overcome selection bias in this sense, we employ sensitivity analysis following the approach of Nannicini [28]. In addition, we check the self-selection issue based on unobserved characteristics through maximum likelihood estimation of endogenous switching technique [29].

## Results

A simple comparison of means of outcomes confirm the presence of significant difference between participant and non-participant households. However, one can question the non-random nature of participation decision confirmed with significant differences pre-treatment variables across groups. As such, a simple comparison of outcomes without controlling for self-selection might lead to a biased result. After the Logit estimation, age of the household head, community leadership role and livestock ownership in Tropical Livestock Unit (TLU) are found significant to influence the participation decision of the household in the project (see Table 2). This could be an indication for the existence of low level of systematic difference between the participant and non-participant households. The propensity scores based on the Logit estimation are saved for further analysis (common support region, matching algorithm and treatment effect estimation).

**Table 2:** Logit results of households’ project participation.

Variables	Coefficients	Robust Std. err	Z value
Age of the household head	-0.03	0.01	-2.18**
Sex of the head	0.54	0.42	1.33
Community role of the head	1.05	0.36	2.96 ***
Literacy of the household head	0.32	0.36	0.88
Livestock (TLU)	0.09	0.05	1.74*
Family size	0.11	0.08	1.30
Landholding	0.29	0.19	1.50
Irrigated landholding	-0.73	0.94	-0.78
Time to extension off.	0.69	0.50	1.35
Time to frequent market	-0.11	0.32	-0.33
Time to the woreda town	-0.33	0.22	-1.51
Constant	-0.27	0.87	-0.31

Notes:

***, ** and *means significant at the 1%, 5% and 10% probability levels, respectively.

Model summary: Pseudo R^2^ = 0.12, Log likelihood = -122.08, Wald chi^2^ = 25.59, Prob>chi^2^ = 0.00, and 200 observations.

### The common support

A very straight forward criterion to know the common support region is graphical technique, by drawing propensity scores against their frequency density distribution. The common support region then can be identified simply based on the graph, using the propensity scores and the density estimation procedure. The full sample, the participant and non-participant households’ estimated propensity scores are plotted against density estimation (S1 Fig). Looking at the distribution of the propensity scores and the overlaps, one can see that most of the observations lie in the common support region. Accordingly, we can conclude that only few observations can be rejected from the analysis. We can compare this result with the outcomes of the matching algorithm presented in the next section. As one can observe, a range of 167 (in Radius Caliper matching with 0.01 band width) to 190 (in most other matching algorithms) observations are retained for the impact analysis using matching algorithms based on propensity scores.

### Matching algorithm

We employ different matching algorithms for the estimation of the treatment effect. The number of matched observations, the pseudo R-square value and balancing test are the three criterion employed to select the matching algorithm. According to the selection criterion, we select caliper matching with 0.25 band width. This matching algorithm resulted with large matched sample size, lowest pseudo R^2^ value and balanced all the covariates after the rejection of observations outside of the common support region (see Table 3). The balancing test tells us the number of covariates remain balanced after the matching procedure. In other words, there is no significant difference in the mean and/or frequency distribution of the covariates of the participant and non-participant households after the matching procedure. Hereafter, the estimation results of the treatment effect described and discussed throughout the paper are calculated based on caliper matching.

**Table 3:** Performance of matching estimators under the three criteria.

Matching estimator	Performance criteria		
	Balancing test	Pseudo-R^2^	Matched sample size
Neighbor matching			
1 neighbor	10	0.05	176
2 neighbor	8	0.11	190
3 neighbor	7	0.10	190
Radius Caliper matching			
0.01	8	0.06	167
**0.25**	**11**	**0.005**	**190**
0.5	8	0.07	190
Kernel Matching			
With 0.1 band width	8	0.08	190
With 0.25 band width	11	0.02	190
With 0.5 band width	11	0.03	190

### Treatment effect of project participation

We do find an evidence that the project has brought significant improvements on key productivity and market orientation indicators. The result is in line with the premises that the participation of the household in the project can improve market integration, efficiency of farms and the overall gain from farming. We present the treatment effect estimation result in Table 4. The participant households on average employ more purchased agricultural inputs and gain higher gross margin from the production activities as compared to the non-participant households. The non-participant households on average supply less output (measured both in monetary terms and proportion of total produce) to the market as compared to their participant counterparts.

**Table 4:** ATT of the project on market orientation.

Variable	ATE	Std. err	ATE	Std. err
	*Unmatched*		*Matched Sample after bootstrapping*	
Purchased inputs (birr)	4398.99***	660.89	3764.08***	730.42
Market supply of the HH (birr)	9289.4***	1797.67	8192.00***	1979.90
Share of the produce sold	0.168***	0.025	0.157***	0.035
Gross margin	8851.71***	2275.06	7369.06***	2441.45

*** indicates significant at the 1% probability level.

We employ input oriented DEA approach to analyze the technical efficiency of farm households in terms of the production of major food crops (wheat, bean, pepper and potato). The estimated technical efficiency scores are then compared among the participant and control households after the matching procedure. As can be seen in Table 5, there exist technical efficiency difference among farmers in general in the major crops under consideration. Except for the technical efficiency of production in potato, project participant households are better-off in production efficiency compared with the non-participant counterparts.

**Table 5:** ATT of the project on farm efficiency.

Variable	ATE	Std. err	ATE	Std. err
*Technical efficiency*	*Unmatched*		*Matched Sample after bootstrapping*	
Wheat production	0.096***	0.025	0.042*	0.024
Bean production	0.102***	0.039	0.084**	0.036
Pepper production	0.179***	0.033	0.178***	0.041
Potato production	0.114***	0.036	0.056	0.043

Source: Own estimation result.

### Robustness checks

Except a little deviation, we do find consistent results with the original PSM model after the sensitivity analysis. After sensitivity adjustment, the efficiency difference in wheat production among participant and non-participant farmers becomes insignificant. We also check the consistency of results using endogenous treatment switching regression (ESR) technique. Though we confirm significant selection from unobservable characteristics towards project participation in some of the estimations, the overall result and conclusion remain consistent in both approaches (S1 Table). The likelihood ratio test and rho confirm significant correlation between the errors in the treatment and outcome equations in the estimation of gross margin, share of farm product sold and the efficiency estimation of wheat. In the ESR, we do find positive and significant effect of project participation on the use of purchased inputs, market supply and gross income. Likewise, project participants have higher technical efficiency in the production of the major crops.

## Discussion

### Participation in the project

We confirm that age of the household head and community leadership role (either political, social or religious) is among the factors that significantly determine the probability of project participation. Community leadership role is also a particularly relevant variable to determine the participation decision. Community leaders are likely to be more informed, have easy access and are better prepared to join development projects [30, 31]. Caeyers and Dercon [31] found a significant association between vertical political connections of the household and the probability to get food aid. Livestock ownership in TLU is among the variables that determine the propensity of project participation. Livestock is a source of draft power for crop production activities, store of wealth, and risk mitigation mechanism in the crop-livestock mixed agricultural systems. Livestock ownership is often considered as a proxy to the asset holding and wealth of the rural households in Ethiopia [32].

Overall, we found out that some of the explanatory variables considered significantly influence the participation decision of the household. This verifies the non-random nature of the treatment assignment and the issue of self-selection. Hence, analyzing the impact of treatment on outcome variables without controlling for selection bias can lead towards wrong conclusions. As PSM controls for selection bias from observable characteristics, the matching procedure is employed to correct for such a bias.

### Impact of IPMS project participation

The integrated agricultural extension approach by the IPMS project has contributed to the use of purchased inputs, increased income and marketed surplus of the participant households. This result is in line with impact assessment papers on agricultural extension services in the developing world [33–36]. A study in Ghana revealed a significant contribution of the adoption of water conservation and intensification technologies on the productivity and net income of farmers [35]. According to a study conducted in Tigray (North Ethiopia) using Propensity Score Matching method, the new rural extension program implemented in Ethiopia has contributed to household welfare, investment and income diversification [36]. Using matching technique, Pedro, Maffioli [37] reported mixed impact of agricultural extension service in Argentina on yield and quality of grape. Program participation has improved productivity of grape for less productive farmers. On the other hand, middle and large farmers improve the quality of grape after participation [37]. These empirical literatures confirm the impact of agricultural extension programs on the livelihood of the poor in the developing world.

Participation in the project improves the technical efficiency of production of major crops except for potato production. The finding of the study is in line with the research hypothesis and empirical evidences in the developing world. Agricultural extension service by different providers in Uganda brought a significant improvement in the technical efficiency of farm households [38]. Using a panel data set in Ethiopia, Gezahegn, Mekonnen [39] have got mixed results on the contribution of agricultural extension service on technical efficiency of major crops. Extension supported farmers record higher efficiency in wheat production while lower efficiency in *Teff* and Maize production [40]. Another study reported that frequency of contact with the extension agents is negatively associated with efficiency of production [41]. The author argued that the frequency of contact of the extension agents with farm households cannot make a difference unless supported with improved technology and knowledge. Overall, improved skill in operation and linkage input and output market with the participation in the project bring about a significant change in production efficiency, improve farm household’s income and their marketed surplus. Based on our empirical evidence, we argue that multi-faceted and integrated agricultural extension service accompanied with further targeting can improve the impact of the system.

## Conclusion and Policy Implications

Improving Productivity and Market Success (IPMS) of Ethiopian farmers’ project was initiated with the idea of implementing integrated extension approaches in selected pilot learning sites in Ethiopia. Using a cross-section data of 200 households in Bure district, we analyze the impact of the intervention of the project in the use of purchased inputs, marketed surplus, efficiency and income of farm households. We employ PSM approach for impact assessment and further test robustness checks. One of the key intervention areas of the project is to facilitate the use of improved technologies and encourage market orientation in agriculture. We have seen an improvement in the production orientation of farmers towards market oriented production. Based on our analysis, we confirm that the project has contributed for the input and out market integration and/or market oriented agricultural production. This paper confirms the possibility of significant technical efficiency improvement on both the project participant and non-participant households. Farmers participating in the project are better-off in technical efficiency of producing major food crops compared to the control households. This reveals that the pilot project with an integrated agricultural extension approach can improve the production efficiency of farmers in Ethiopia. Overall, we argue that the IPMS project intervention can be seen as an experimental model with a promising potential to improve the livelihood of the poor. Furthermore, we suggest that it is worthwhile to employ integrated agricultural extension programs with further targeting in the developing world. Finally, as PSM only controls treatment selection from the observable characteristics of the household, the results should be interpreted with caution.

## Supporting Information

S1 Fig Kernel density of propensity scores. (TIFF)

S1 Table Robustness checks with endogenous switching regression technique. (PDF)
