# Supplementary figures and images for: Production Efficiency and Market Orientation in Food Crops in North West Ethiopia: Application of Matching Technique for Impact Assessment
Source: PLoS One. 2016 Jul 8;11(7):e0158454. doi: 10.1371/journal.pone.0158454 (PMC4938615; doi:10.1371/journal.pone.0158454)

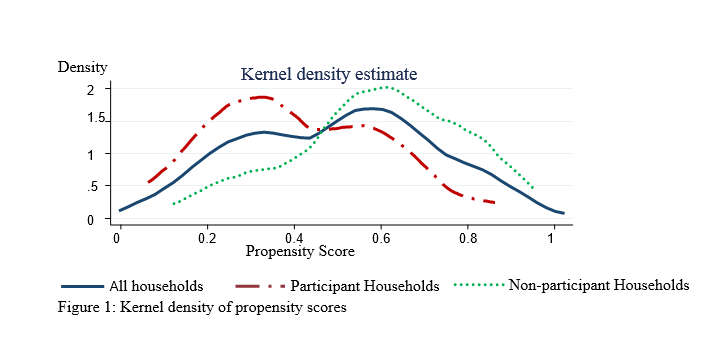

Supplement: S1 Fig — (TIFF) [file pone.0158454.s001.tiff]
