# Supplementary material for: Production Efficiency and Market Orientation in Food Crops in North West Ethiopia: Application of Matching Technique for Impact Assessment
Source: PLoS One. 2016 Jul 8;11(7):e0158454. doi: 10.1371/journal.pone.0158454 (PMC4938615; doi:10.1371/journal.pone.0158454)
Supplement: S1 Table — (PDF) [file pone.0158454.s002.pdf]

Appendix 1: Robustness checks with endogenous switching regression technique

| Model | Treatment outcomes | | | | | | | |
|---|---|---|---|---|---|---|---|---|
| summary | 1 | 2 | 3 | 4 | 5 | 6 | 7 | 8 |
| Coeff. | 4085*** | 15608*** | 0.261** | 13799*** | 0.285*** | 0.255** | 0.368*** | 0.060 |
| (Std. err) | (1291) | (1853) | (0.107) | (2589) | (0.010) | (0.101) | (0.104) | (0.122) |
| rho | -0.397 | -0.798*** | -0.460 | -0.663*** | -0.782*** | -0.481 | -0.646 | 0.069 |
| | (0.266) | (0.063) | (0.329) | (0.097) | (0.089) | (0.237) | (0.216) | (0.403) |
| LR test Chi2 | 0.82 | 18.85*** | 1.07 | 9.39*** | 8.63 | 2.04 | 2.06 | 0.03 |
| (Prob > chi2) | (0.37) | (0.00) | (0.30) | (0.00) | (0.00) | (0.15) | (0.15) | (0.87) |

*Note: 1=Purchased inputs (birr), 2= Market supply of the HH (birr), 3=Share of the produce sold, 4= Gross margin, 5= Technical efficiency in wheat production 6= Technical efficiency in bean production, 7= Technical efficiency in pepper production and, 8= Technical efficiency in potato production*
